# Intense Habitat-Specific Fisheries-Induced Selection at the Molecular *Pan* I Locus Predicts Imminent Collapse of a Major Cod Fishery

**DOI:** 10.1371/journal.pone.0005529

**Published:** 2009-05-27

**Authors:** Einar Árnason, Ubaldo Benitez Hernandez, Kristján Kristinsson

**Affiliations:** 1 Institute of Biology, University of Iceland, Reykjavík, Iceland; 2 Institute of Biology, University of Iceland, Reykjavík, Iceland; 3 Marine Research Institute, Reykjavík, Iceland; University of Utah, United States of America

## Abstract

Predation is a powerful agent in the ecology and evolution of predator and prey. Prey may select multiple habitats whereby different genotypes prefer different habitats. If the predator is also habitat-specific the prey may evolve different habitat occupancy. Drastic changes can occur in the relation of the predator to the evolved prey. Fisheries exert powerful predation and can be a potent evolutionary force. Fisheries-induced selection can lead to phenotypic changes that influence the collapse and recovery of the fishery. However, heritability of the phenotypic traits involved and selection intensities are low suggesting that fisheries-induced evolution occurs at moderate rates at decadal time scales. The Pantophysin I (*Pan* I) locus in Atlantic cod (*Gadus morhua*), representing an ancient balanced polymorphism predating the split of cod and its sister species, is under an unusual mix of balancing and directional selection including current selective sweeps. Here we show that *Pan* I alleles are highly correlated with depth with a gradient of 0.44% allele frequency change per meter. *AA* fish are shallow-water and *BB* deep-water adapted in accordance with behavioral studies using data storage tags showing habitat selection by *Pan* I genotype. *AB* fish are somewhat intermediate although closer to *AA*. Furthermore, using a sampling design covering space and time we detect intense habitat-specific fisheries-induced selection against the shallow-water adapted fish with an average 8% allele frequency change per year within year class. Genotypic fitness estimates (0.08, 0.27, 1.00 of *AA*, *AB*, and *BB* respectively) predict rapid disappearance of shallow-water adapted fish. Ecological and evolutionary time scales, therefore, are congruent. We hypothesize a potential collapse of the fishery. We find that probabilistic maturation reaction norms for Atlantic cod at Iceland show declining length and age at maturing comparable to changes that preceded the collapse of northern cod at Newfoundland, further supporting the hypothesis. We speculate that immediate establishment of large no-take reserves may help avert collapse.

## Introduction

Predation is a powerful agent in the ecology and evolution of predator and prey. Prey may select multiple habitats whereby different genotypes prefer different habitats. If the predator is also habitat-specific the prey may evolve different habitat occupancy. Drastic changes can occur in the relation of the predator to the evolved prey. For example the predator may exterminate the prey in the habitat available to the predator and thus lose its prey. The prey selecting the alternative habitat may be released from predation and might even evolve to become a new species free of predation. The stronger the predation and more efficient the predator, the more drastic would be the evolutionary changes. It is, therefore, important to study the way predation acts on genes in species that select multiple habitats by genotype.

Man the hunter has become a mechanized techno-beast, a highly efficient predator. In particular, commercial fisheries searching for fish with computerized fish-finders and airplanes and scooping up fish with several thousand-ton capacities with ships powered by several thousand horsepower engines are a case in point [1]. Modern fisheries are uncontrolled experiments in evolution [2], [3]. Fisheries target certain phenotypes and, therefore, can be a powerful agent of natural selection. They also frequently are habitat-specific by concentrating on the most accessible locations.

Fisheries-induced selection [4]–[10] can lead to phenotypic changes that influence the collapse and recovery of fisheries [4], [5], [11]. Fisheries-induced selection is primarily discussed as selective mortality directly targeting certain phenotypes such as length- or weight-at-age [4], [6], [7] by size-selective fishing or indirectly targeting phenotypes such as age-at-maturation due to age-at-entry into the fisheries or by location of fishery [4]. However, the genetic determination and heritability of the quantitative traits involved is largely unknown [4], [8], [12]. General estimates of the heritability of life-history traits are low as are estimated selection intensities although fishing mortality can be very high [13], [14]. From these observations emerges the view that fisheries-induced evolution occurs at moderate rates with significant changes only observable at decadal time scales [13]. Nevertheless, the recommendation is that the management adopt short-term conservation/management measures that also meet concerns about long-term evolutionary impact. Thus conservation of old, big fish is promoted as a combined short- and long-term conservation strategy [13]. Evolutionary changes were implicated in the collapse and non-recovery of the northern cod of Newfoundland and in the near collapse of North Sea cod [11], [13], [14]. Following the collapse of the northern cod fishery in Newfoundland [11], the cod fishery at Iceland has remained as one of the worlds major and ostensibly sustainable cod fishery.

The single molecular Pantophysin I (*Pan* I) locus represents an ancient balanced polymorphism predating the split of Atlantic cod (*Gadus morhua*) and its sister species [15]. The *A* and *B* alleles at the locus differ by multiple nucleotide and six amino-acid substitutions. The locus exhibits local adaptation and is under an unusual mix of balancing and directional selection including current selective sweeps [16], [17]. *Pan* I variation also has been taken as evidence for population sub-structuring caused by breeding structure [18], [19]. However, the precise roles that fisheries-induced selection and natural selection play in generating that structure are unknown.

Here we test the hypothesis that fisheries can exert powerful habitat-specific selection on a fish species with consequent evolutionary changes. We report exceptionally strong selective changes at the *Pan* I locus which is involved in habitat selection of depth by genotype in populations of Atlantic cod at Iceland. With a heritability of 100% and indirect but intense selection due to fishing in preferred habitat we observe significant changes on a yearly basis. Thus, fisheries-induced evolution is short term [20]. We identify a threat of collapse due to the selection imposed by the fishery. We consider that if management acted immediately it may be possible to avert collapse. Our study demonstrates the importance of molecular population genetics of targeted loci for studies of fisheries-induced selection and highlights the importance of evolutionary thinking for both short- and long-term management of exploited fish populations.

## Results

### Allele frequency and depth of sampling

**Figure 1:**
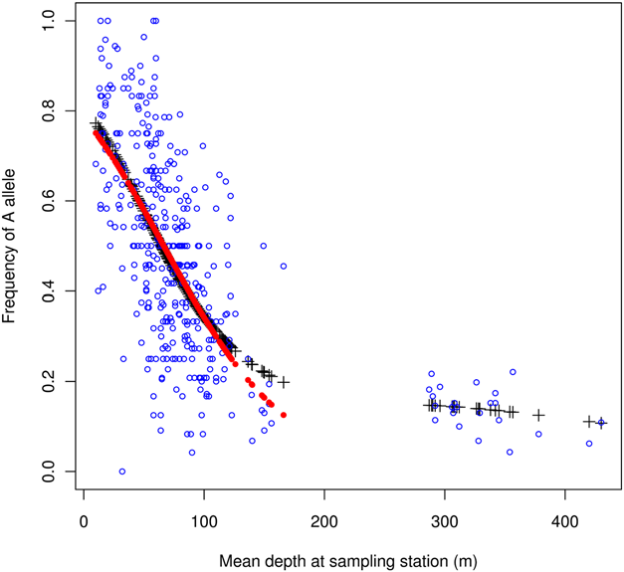
Frequency of *Pan* I *A* allele on mean depth (m) of sampling. Points (open circles ○) represent frequency at all sampling stations for Atlantic cod in Icelandic Marine Research Institute spring spawning surveys in 2005, 2006, and 2007. Pluses+represent a generalized additive model (gam) smooth fit. Solid dots • represent a generalized linear regression (glm) of allele frequency on depth for depths less than 200 m; glm linear predictor *η* = 1.297−0.0195depth yields an allele frequency intercept of 78.5% and 34.2% at 100 m, a 44.3% change.

The relationship of allele frequency and depth was highly regular and significant (Figure 1, Table 1 and Figure S1; and see Table S1 for an overview of supplementary data) with frequency of the *A* allele decreasing rapidly to a depth of less than 200 m but staying relatively level in deeper waters. A linear regression equation of allele frequency of *A* on depth for depth less than 200 m was *P_A_* = 0.806−0.0044*D* (*t* = −14.6, *P*≪0.001) or a 0.44% drop in allele frequency per meter. A generalized linear model (glm) fit gave almost identical results (Figure 1).

**Table 1:** *Pan* I allele frequencies and Hardy-Weinberg deviations by 25 m depth classes among Atlantic cod.

	Spring, spawning				Fall, feeding			
Depth	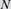	*p_A_*	*F*_IS_	*X* ^2^	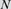	*p_A_*	*F*_IS_	*X* ^2^
0–25	345	0.759	0.175	10.57**				
25–50	691	0.656	0.237	38.91***	8	0.875	−0.143	0.16
50–75	1824	0.500	0.129	30.54***	107	0.813	0.139	2.07
75–100	1187	0.393	0.041	2.01	166	0.699	−0.059	0.58
100–125	521	0.315	−0.041	0.88	50	0.640	−0.302	4.56*
125–150	170	0.159	−0.101	1.73	270	0.443	−0.103	2.89
150–175	83	0.289	0.121	1.21	171	0.447	−0.053	0.47
175–200					179	0.243	−0.048	0.41
200–225					182	0.209	0.035	0.23
225–250					283	0.269	−0.007	0.02
250–275					127	0.323	−0.117	1.73
275–300	139	0.169	0.053	0.38	118	0.288	−0.074	0.65
300–325	129	0.163	−0.024	0.07	209	0.220	−0.087	1.59
325–350	178	0.140	−0.163	4.75	261	0.276	−0.189	9.48**
350–375	46	0.120	−0.136	0.85	84	0.286	−0.050	0.21
375–400	24	0.083	−0.091	0.20	153	0.320	−0.111	1.88
400–425	24	0.062	−0.067	0.11	168	0.196	−0.169	4.80*
425–450	23	0.109	−0.122	0.34	145	0.231	−0.068	0.66
450–475					10	0.350	−0.099	0.10
475–500					84	0.244	−0.258	5.61*
500–650					37	0.243	−0.321	3.82*
Sum	5384	0.444	0.195	205.28***	2812	0.342	0.040	4.60*

Atlantic cod at spring spawning and fall feeding grounds at Iceland. Number of individuals, *N*, and significant deviations from Hardy Weinberg represented by starred *X* ^2^ statistics: *: *P*<0.05; **: *P*<0.01;***: *P*<0.001. Frequency of *A* allele: *P_A_*. Deviation from Hardy-Weinberg equilibrium: *F*_IS_. Test statistic: *X* ^2^.

In spring, significant heterozygote deficiency characterized the three top 25 m depth layers, an apparent Wahlund effect [21] due to the convergence in shallow waters of groups of fish that differed in allele frequencies. *AA* fish were rare in deep water in spring but were found at all depths in fall although they preferred shallow water (Table 1). In fall significant deviations from Hardy-Weinberg were found at various depths representing heterozygote excess in all instances. For fall and spring combined the sign of *F*_IS_ was most often negative (sign test, *P*<0.01) indicating a general tendency for heterozygote excess.

### Population differentiation in a two and a three dimensional habitat

**Figure 2:**
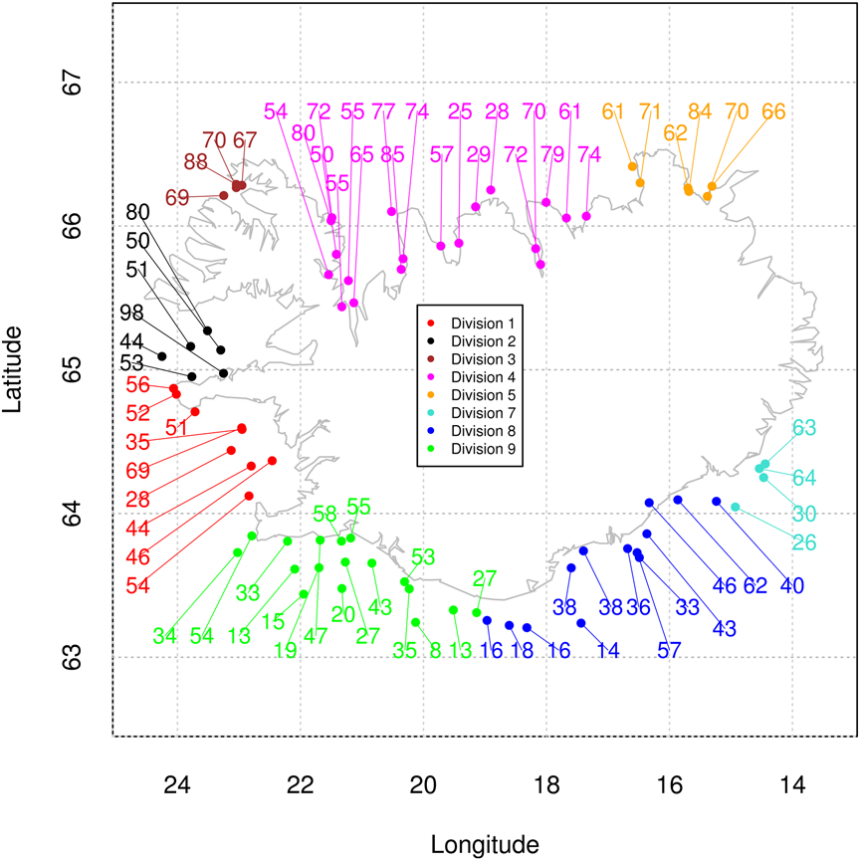
Frequency (percent) of the *Pan* I *A* allele in squares within areas. Areas defined by one degree longitude and one half degree latitude (dotted lines) are each split into four equal sized squares (not shown). Sampling stations within subareas are pooled for frequency estimation. Atlantic cod in Icelandic Marine Research Institute spring spawning surveys in 2005, 2006, and 2007. Color coded divisions based on revised metacod definitions as detailed in paper [19], [61].

Spatial population differentiation exhibited by *Pan* I [19] is more fully explained by differences in depth among localities although depth and locality are confounded. We found large differences in allele frequencies among localities defined by pooling sampling stations in squares within areas (Figure 2; we pooled to increase sample size). Close inspection of the figure, however, shows that there can be large allele frequency differences among neighboring localities within divisions. These may be described as an inshore/offshore difference, however, depth is a more important explanatory variable. We found apparent spatial differentiation significant in all instances (*P* = 0.001 based on 1000 permutations in all instances. Table 2). Interestingly, the overall *F*_ST_ = 0.074 was considerably lower than found for Northeast-Southwest comparison [19]. Also the differentiation among sampling stations within divisions was higher than among divisions (Table 2).

**Table 2:** Hierarchical *F* statistics among divisions, among sampling stations within divisions and among individuals within stations.

	Division	Station	Individual
Total	0.074	0.192	0.208
Division		0.128	0.145
Station			0.020

Significance *P* = 0.001 based on 1000 permutations in all instances.

Considering depth of sampling there is clearly a confounding of depth and geographic location. Shallow stations are located in the Northeast and North whereas very deep-water stations are exclusively in the South and Southwest (Figure S2 and Figure S3). Thus, the greatest contrast in depth was between the Northeast and South/Southwest. Given the gradient of 0.44% change in allele frequency with 1 m change in depth (Figure 1 and Figure S1), depth differences between the Northeast and South/Southwest very likely contributed to the apparent spatial differentiation of Icelandic cod [19]. There were in some instances large differences in allele frequencies among neighboring stations within divisions (Figure 2) particularly in divisions that included sampling stations of different depths (Figure S3). Because depth and location are essentially a factorial or crossed design [22] we cannot use hierarchical or nested *F*-statistics to test their effects. However, a crossed factor can be tested independently within a level of the other factor [23] and probabilities combined in meta analysis [24]. Differentiation by depth within divisions (Table 3) was relatively high and significant in all instances except division 3. The test combining probabilities was also significant (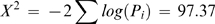, *df* = 16, *P*≪0.001). In contrast, considering divisions within depth classes differentiation was considerably lower (Table 4) but nevertheless significant in several instances and overall (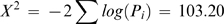, *df* = 22, *P*≪0.001).

**Table 3:** *F* statistics among depth classes within divisions.

Division	*F*_Depth/Tot_	*F*_Ind/Tot_	*F*_Ind/Depth_	*df*_D/T_	*df*_I/D_	*df*_I/T_	*G*	*P*
Division 1	0.048	0.146	0.103	3	505	509	30.98	0.001
Division 2	0.100	0.114	0.015	5	783	789	137.81	0.001
Division 3	−0.012	0.051	0.062	2	112	115	0.80	0.717
Division 4	0.048	0.203	0.162	5	542	548	49.21	0.001
Division 5	0.053	0.138	0.090	1	333	335	14.02	0.001
Division 7	0.263	0.153	−0.149	3	136	140	67.43	0.001
Division 8	0.126	0.087	−0.045	9	996	1006	232.07	0.001
Division 9	0.137	0.242	0.121	10	1931	1942	431.75	0.001
Overall	0.135	0.217	0.095	13	5370	5384	677.39	0.001

Statistics of Depth in Total, Individuals in Total and Individuals in Depth. *G* is test statistic and *P* is based on 1000 permutations in all instances. Depth classes are the same as in Table 1 and Table 4.

**Table 4:** *F* statistics among divisions within depth classes.

Depth Class	*F*_Div/Tot_	*F*_Ind/Tot_	*F*_Ind/Div_	*df*_D/T_	*df*_I/D_	*df*_I/T_	*G*	*P*
0–25	0.090	0.219	0.142	2	342	345	40.33	0.001
25–50	0.019	0.224	0.209	7	585	593	31.70	0.002
50–75	0.026	0.149	0.127	6	1881	1888	75.37	0.001
75–100	0.054	0.048	−0.006	6	1190	1197	104.40	0.001
100–125	0.058	−0.008	−0.070	4	528	533	50.68	0.001
125–150	0.060	−0.055	−0.122	2	134	137	9.50	0.007
150–175	0.323	0.293	−0.045	1	126	128	35.22	0.001
275–300	−0.007	0.053	0.059	1	137	139	0.01	0.866
300–325	−0.007	−0.025	−0.018	1	127	129	0.45	0.515
325–350	0.006	−0.156	−0.163	1	176	178	1.74	0.189
350–375	0.088	−0.077	−0.180	1	44	46	5.42	0.033
Overall	0.077	0.208	0.142	7	5376	5384	677.39	0.001

Statistics of Division in Total, Individuals in Total and Individuals in Division. *G* is test statistic and *P* is based on 1000 permutations in all instances. Divisions are the same as in Table 3.

Overall, therefore, there was greater differentiation by depth than by geographic locality but the two factors remain statistically confounded. Depth of course is a proxy for some biological and environmental factors [25]. For the purposes of this paper the relationship with depth is of great importance. Even if significant spatial variation existed that was not confounded by depth it would not alter implications of the selective effects discussed here.

### Fishing pressure

Data on Atlantic cod catches show that, in general, the brunt of the fishing is carried by 5–7 year old fish. By eight years of age a year class is severely reduced and by nine years it is all but fished out (Figure S4; and see Table S1 for key points of supplementary data). Furthermore, analysis of catch by gear shows that the heavy fishing occurs in shallow water (Figure S5). Catch by long line is to some extent conducted in deeper water (200–300 m) with a shift towards shallower waters in recent years as shown by the higher density of catch at depths less than 100 m and lower density at 200–300 m depths (Figure S5). Although also taking considerable catch in shallow waters, fishing by bottom trawl is distributed over the greatest range of depth (Figure S5).

Bottom trawl which targets greatest depth range brings in more than 40% of the catch (Figure S6). From 1997 to 2007 the catch taken by gear targeting shallow water has increased. Catch with hand line, Danish seine, and gill nets increased up until 2003. However, their catch have decreased since then. Catch with bottom trawl has decreased but catch with long line has increased substantially and after 2005 nearly matches the catch taken by bottom trawl. Thus, fishing mortality is heaviest in shallow water [26] and at least after 2000 fishing targeting shallow water has increased.

Total catch is regulated by government issued quotas of total allowable catch (TAC) which are based on advice from the Marine Research Institute (MRI) of recommended total allowable catch (recommended TAC). Current management strategies of Icelandic cod are based on a catch limitation system where each vessel is allowed a certain share of the TAC. Annual recommended TACs are based on scientific assessment of state of fish stocks and ecosystem condition, but have been reduced lately. For example, the TAC for the 2007/2008 fishing year was 130,000 tons, a 63,000 ton reduction from the previous year. The recommended TAC for the following fishing year was a further 7000 ton reduction but 160,000 was issued. There is a tight correlation of total catch, issued TAC, and recommended TAC. However, total catch almost invariably exceeds issued TACs which in turn are always greater than recommended TAC except for 1996 to 2007 when a catch rule has been in effect (Figure S7).

**Figure 3:**
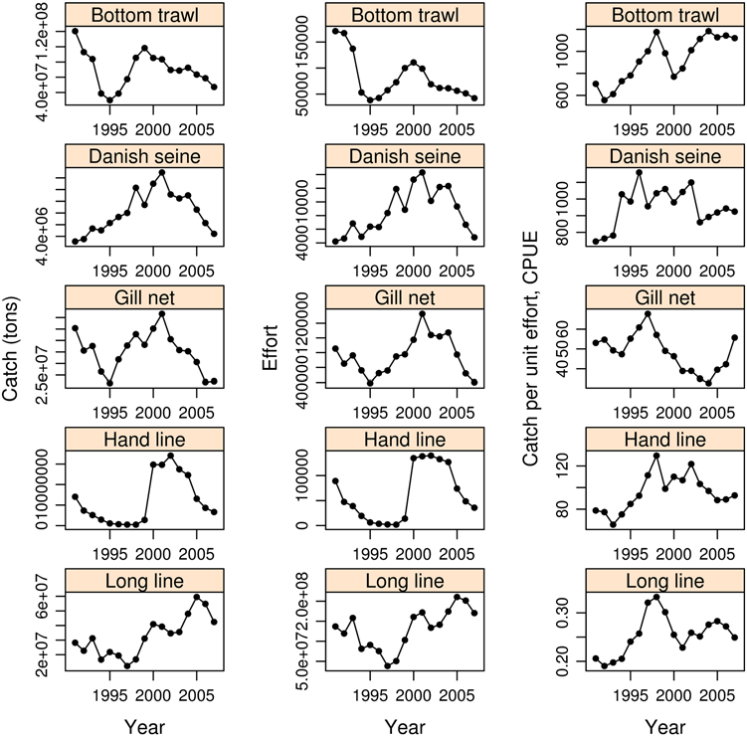
Catch (tons), effort and catch per unit effort, CPUE, at year for different gear. Data are from log book records. Parts of these data are the same as figures 9.3.1. and 9.3.2 in [62]. Units of effort for different gear are described in Methods.

Annual catch, effort, and catch per unit effort for different gear show a complex interaction with each other and with quotas and TACs (Figure 3 and Figure S7). The stock reached an all time low population numbers in 1993–1995 [27] (Figure S7). In the following years the MRI estimated an increase in numbers and increased quotas were issued. Catches of most gear increased and increased effort followed immediately or with a lag (of up to one year) for some gear. Both catch and effort peaked around 2000 and 2001. Catch per unit effort peaked earlier. Catch and effort of both long line and hand line started to increase later than other gear and peaked later or in 2004 and decreased somewhat after that (Figure 3). This, at least partly, accounts for the relative increase of catch by long line in the total catch (Figure S6). As catch diminished after reaching a peak, effort was reduced more rapidly and thus the relative measure of catch per unit effort increased. Using the relative measure of catch per unit effort as an indicator of stock abundance must take total catch into account and whether it is increasing or decreasing [28].

Following the 1995 crash government issued a new catch rule limiting annual quotas to 25% of average fishable biomass. In 2000 and again 2001, with the benefit of hindsight, the MRI re-estimated the population numbers for the previous years. A considerable stock size overestimation and underestimation of fishing mortality was apparent [29], [30], amounting to 25–50%. Issued TACs were based on the overestimated stocks and catches have been 27–40% of the fishable stock, far exceeding the target of 25%. Thus, for example, for 2000 fishing mortality was estimated at 0.86 compared to approximately 0.4 if the catch had been at the 25% target [30]. Consequently the population has experienced increased predation pressure through increased fishing mortality [31].

Part of the overestimation is explained by a lower than predicted mean weight at age [30]. Changes in catchability [28], [31] are, however, the main explanation [30]. There were also changes towards fishing older and larger fish. As an example the gill net fleet changed most of its nets from 7 inch to 9 inch mesh size from 1994–1998 [30].

### Probabilistic maturation reaction norms

**Figure 4:**
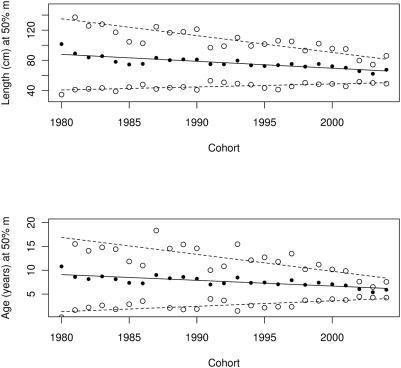
Probabilistic maturation reaction norms: length and age at 50% probability of maturing on cohort. Solid dots and solid lines represent length or age at 50% probability of maturing. Upper and lower open dots and dashed lines represent length or age at 95 and 5% probability of maturing respectively. Lines are linear regression of length or age on cohort. Based on data on mean length, age and maturity ratio from table 3.1.4 in Anonymous [27] (and see [74]).

We applied the principles of estimating probabilistic maturation reaction norms [32]–[34] to evaluate the potential effects of fisheries on changes in life-history traits. We used data on mean maturity, length and age [35] and estimated maturity ogives. From these we estimated the probability of maturing *m* at length or age (Figure 4). Quantiles of length (and age) at both 50% and 95% probability of maturing show a significant downward trend (slope of linear regression: −0.9 and −2.2 cm per year respectively, *P*≪0.001). Length (and age) at 5% probability of maturing increased (slope of linear regression: 0.4 cm per year, *P*≪0.001).

### Genic and genotypic frequencies at age

**Figure 5:**
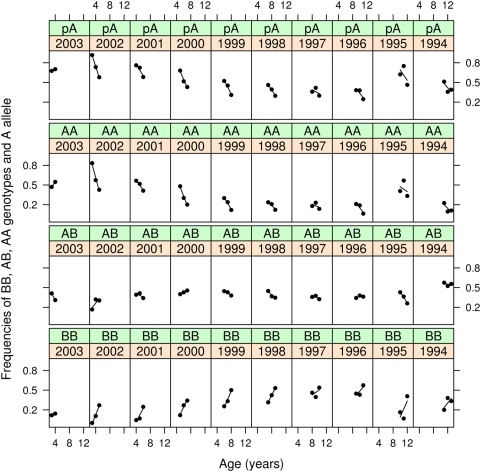
Allelic and genotypic frequencies at age conditioned on year class. Spring spawning Atlantic cod at Iceland. Frequency of *A* allele, *p*_A_ (top panel row), and frequencies of *AA*, *AB*, and *BB* genotypes, *f_AA_*, *f_AB_*, and *f_BB_* (panel rows 2–4 respectively). Panels represent year classes arranged most recent to older from left to right in each row. Points • represent observed frequencies; lines represent linear regression of frequency on age.

There were large and highly regular allele and genotypic frequency changes on age within year classes (Figure 5 and Table 5). Year classes are independent realizations of birth and death processes yet changes were in the same direction. Allele and genotypic frequencies in older year classes appeared as continuation of frequencies among more recent year classes (Figure 5). Comparing the 2002 to 1996 year classes, frequencies of *A* allele on age decreased both within and among year classes (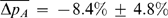 per year). Similarly, frequencies of *AA* decreased (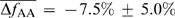), *AB* increased at young ages but decreased slightly on average (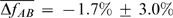), and *BB* increased (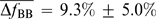). There was a reversal with *BB* becoming the most common genotype by about eight years of age. The 1995 and 1994 year classes had higher *A* allele frequencies compared to the more recent year classes. However, comparing these two year classes the same pattern of decrease of *AA* and increase of *BB* with age held. Evolutionary changes are observable on a yearly basis [13] and thus ecological and evolutionary time scales are congruent.

**Table 5:** ANOVA table of change of genotypic frequency per year within year class.

Source	Df	SS	MS	*F*	*P*
Genotype	2	0.28	0.14	16.33	<0.00001
Residuals	54	0.46	0.01		

ANOVA table of change of genotypic frequency per year within year class among the *AA*, *AB*, and *BB* genotypes of the *Pan* I locus in Atlantic cod.

We sampled three consecutive years and thus, except for the most recent and the oldest year classes, a year class entered our samples at three ages. Adjacent year classes have two overlapping age classes. The large observed evolutionary changes predict that the *A* allele would be decreasing in frequency. This is exactly what we observed as can be seen by comparing allele frequencies at age (Figure 5 and Figure S8). In general allele frequencies at age are lower among the more recent year classes with a negative slope of a regression of allele frequency at age on year class (Figure S8). This shows that *A* allele frequencies have decreased with time.

### Fitness estimation and prediction of changes

**Figure 6:**
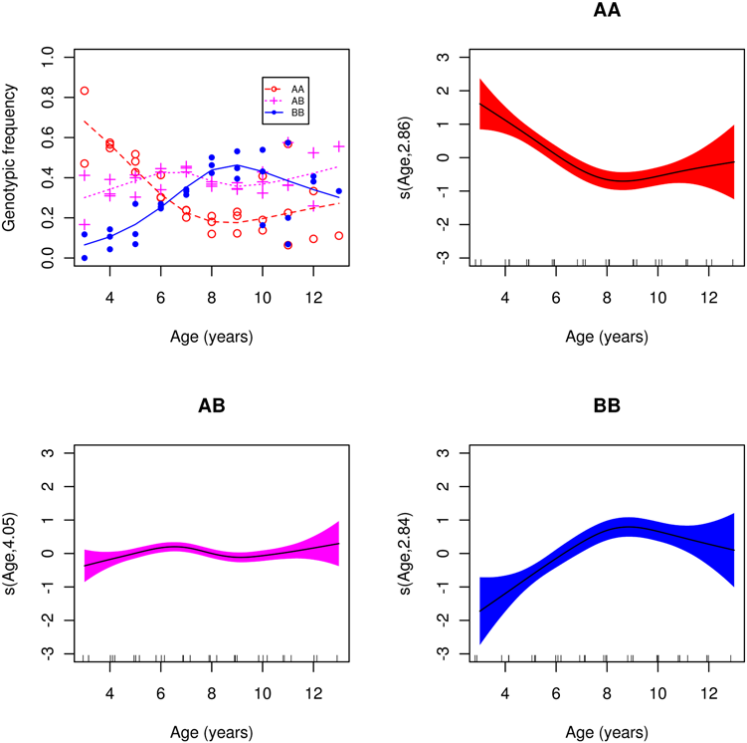
Genotypic frequencies on age in years within year class. Frequencies of *AA* genotype (red open circles ○, dashed line), *AB* (magenta pluses +, dotted line), and *BB* (blue filled circles •, solid line). Lines represent a generalized additive model (gam) smooth fit with quasibinomial link (panel A). Panels B, C and D: gam smooth fit of genotypic frequency on age within year class for the *AA*, *AB* and *BB* genotypes respectively; shaded region represents two standard errors above and below fit. Smooth carries estimated degrees of freedom.

Genotypic frequencies changed significantly between years within year class (Table 5). Frequency changes can be used to estimate relative fitness of *Pan* I genotypes. Overall, the genotypic frequencies changed rapidly to about eight years of age (Figure 6) and stayed relatively level after that, an age at which the brunt of fishing of a year class is over. Catch-at-age data show that by eight years of age, a year class is severely reduced and by nine years is almost fished out (Figure S4). Therefore, the selective pressure of the fishery is mostly over by these ages. Taking notice of this fact we took the ratio of the gam predicted frequencies (Figure 6) among 8 year old (post-selection) to 4 year old (entering the fishery) as weights to estimate fitness (Table 6). Relative *AA* fitness is only 8% and *AB* 27% showing partial dominance. We used the upper and lower confidence limits to predict best-case and worst-case scenarios. Similar low fitness was obtained using slightly different methods for estimation (Table S2 and Table S3).

**Table 6:** Predicted genotypic frequencies, weights and fitnesses.

	*AA*			*AB*			*BB*		
Age 4	lower	esti-	upper	lower	esti-	upper	lower	esti-	upper
Age 8	upper	mate	lower	upper	mate	lower	upper	mate	lower
4 years	0.44	0.57	0.68	0.28	0.34	0.41	0.06	0.11	0.19
8 years	0.21	0.18	0.15	0.41	0.38	0.36	0.48	0.44	0.40
*U_i_*	0.48	0.32	0.22	1.44	1.12	0.88	8.68	4.15	2.06
*W_i_*	0.23	0.08	0.03	0.70	0.27	0.10	1.00	1.00	1.00

Genotypic frequencies of 4 and 8 year old predicted by the gam model in Figure 6. Upper and lower are frequencies ±2 standard errors. Weights, *U_i_*, are ratios of frequencies among 8 year old to 4 year old. Ratios of upper to lower and lower to upper frequencies are used for predictions of best-case and worst-case scenarios respectively. The table is arranged accordingly with lower to upper and upper to lower for each genotype. Fitnesses, *W_i_*, are weights scaled to the most fit *BB* genotype.

**Figure 7:**
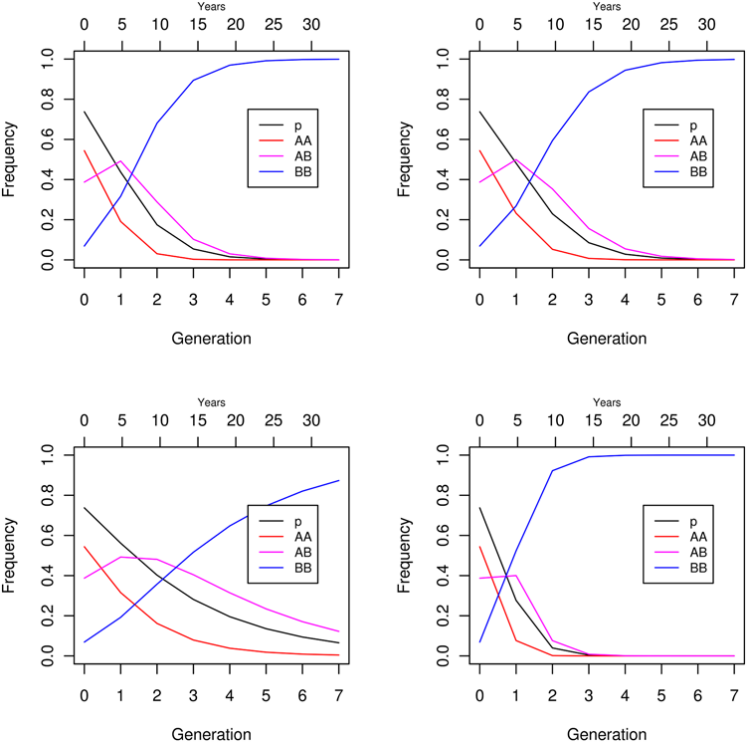
Predicted allele and genotypic frequency changes with a constant-fitness viability model of selection. Top left panel based on fitness estimates from Table 6; top right panel based on fitness estimates from Supplementary Table S2; lower left panel based on highest fitness estimates from Table 6 (a best case scenario); lower right panel based on lowest fitness estimates from Table 6 (a worst case scenario). Starting frequency of 0.738 assumed based on intercept of gam fit in Figure 6. Years based on a generation time of 4.8 years [36]. Color codes are black for the *A* allele and red, magenta, and blue for the *AA*, *AB*, and *BB* genotypes respectively.

Plugging the fitness estimates (Table 6) into an equation for allele frequency change under a constant-viability selection model showed that the *A* allele would be eliminated in 4–5 generations, assuming continued selection of this magnitude. Given a generation time in Atlantic cod of 4.8 years [36], this gives two generations or about 10 years until predicted near disappearance of shallow-water adapted *AA* fish and four generations or 20 years until disappearance of heterozygous *AB* fish (Figure 7 panel 1). Fitness estimates from Table S2 gave similar results (Figure 7 panel 2). Using the most optimistic fitness values (Table 6) doubled the time in a best-case scenario (Figure 7 panel 3) with *AB* heterozygotes still making up some 15% of the population after seven generations. Under a worst-case scenario the most pessimistic values (Table 6) predicted disappearance of *AA* in one generation and of *AB* in two generations or 10 years (Figure 7 panel 4). Similar results (not shown) were obtained with the fitness estimates in Table S2 and Table S3 obtained in slightly different ways.

## Discussion

### Depth and population differentiation

There is an apparent spatial differentiation of Atlantic cod in Icelandic waters with differentiation between the Northeast and Southwest at *F*_ST_ = 0.261 and *F*_ST_ = 0.003 for the *Pan* I and nine microsatellite loci respectively [19]. It is important to examine whether spatial differentiation contributes to the observed selective patterns. The differentiation is clearly driven by the *Pan* I locus. The very low (but significant) differentiation observed at the microsatellite loci is of questionable biological significance [37] especially considering the fact that two of the nine microsatellite loci used (Gmo8 and Gmo34) are outliers in genetic differentiation [38], [39] being influenced either by direct or hitch-hiking selection. In fact, in a study in northern Norway Gmo34 shows strong linkage disequilibrium with *Pan* I [39]. The *Pan* I locus is generally acknowledged to be under selection [15]–[18], [40], [41]. A differentiation of *Pan* I similar to the Icelandic case [19] also is observed for Arctic and Coastal cod in Norway [18]. Both studies [18], [19] concluded that breeding structure with reduced gene flow was the most likely explanation for the observed spatial differentiation, yet both noticed a relationship of *Pan* I allele frequency and depth of sampling similar to the above (Figure 1 and Figure S1).

The apparent Wahlund effects (deficiency of heterozygotes) in shallow water in spring could be interpreted as signs of population structure by depth similar to that observed between coastal and Arctic cod in Norway [18]. To the extent that such segregation by depth actually occurs, the fisheries-induced habitat-specific selection discussed further below would be more efficient in removing shallow-water adapted fish and the effect would be harder to reverse. Conversely, if, as we suggest, there is near panmixia on breeding grounds, selection would be operating in the face of free gene flow between niches. This would reduce the efficiency of selection, it would take longer to lose the shallow-water adapted fish, and the effect would not be as difficult to reverse. A counter argument is based on the observed heterozygote excess at some depths. Overdominance in fitness is one possible explanation for heterozygote excess. However, it is very difficult to detect selection as a deviation from Hardy-Weinberg equilibrium [42]. If fitnesses form a geometric series genotypic frequencies will be in Hardy-Weinberg before and after selection [43] and directional selection may give rise to spurious overdominance if sampling is done before selection is complete [44]. If overdominance was strong enough to produce a significant heterozygote excess we would expect to see it in our fitness estimation. Such strong overdominance would also be expected to counter the heterozygote deficiency observed in spring. Unequal allele frequencies among male and female parents is another textbook explanation for heterozygote excess [45]. But neither overdominance nor male/female allele frequency differences are satisfactory explanations for the observed patterns for it is hard to see why significant excess would be restricted to certain depths at certain times of year. Negative assortative mating is another textbook explanation for heterozygote excess. The intense fisheries-induced selection observed might lead to secondary selection for apostatic mating that would reduce the effects of the selection among their offspring. Thus there could be an advantage for shallow-water adapted phenotypes to be attracted to phenotypes from the opposite deep-water habitat. Instead we consider the generation of heterozygote deficiency in spring (apparent Wahlund effect) and its disappearance in fall as well as the generation of heterozygote excess in fall and its disappearance in spring as a sign of a dynamical system. We consider the patterns most likely to be due to behavioral responses [46]. Fish fitted with data-storage tags can be classified into deep-water and shallow-water behavioral types that either forage and stay mainly in shallow water or migrate to deeper and colder waters foraging at thermal fronts and going to shallow water only for breeding [47]. These types are correlated with *Pan* I: *AA* are shallow-water type, *BB* are deep-water type that move to shallow waters during breeding and *AB* are somewhat intermediate although closer to *AA* than *BB* [46]. *Pan* I genotypes thus are functionally related to behavioral types that select their habitat by depth. Under natural conditions the polymorphism may be balanced by shallow-water vs. deep-water niche-variation specialization. If heterozygous *AB* fish move between the shallow-water and deep-water habitats to a greater extent than homozygous *AA* and *BB* fish there could be at any time an excess of heterozygotes at any depth. However, the role of various mating and feeding behaviors in generating heterozygote excess remains to be studied.

### Selection at *Pan* I

The mechanism of balancing selection at *Pan* I under natural conditions is not known [16] but we suggest specialization to shallow and deep-water niches with free gene flow between the niches as the fish converge on the shallow water breeding grounds. The fitness estimates and relationships observed here, however, very likely tip the balance [16], [17] leading to directional selection in favor of *BB* deep-water adapted fish. It is possible that some unknown fitness component would overcome the mortality leading to overdominance and balanced polymorphism but there is no evidence for that in our results. The selective pressure is most certainly due to habitat-specific fishing mortality that is heavily directed against shallow water fish [26] (Figure S5). If it continues unabated shallow-water fish will disappear rapidly with consequent collapse of the population and the fishery.

We have not considered here the effects of selection and age-structure [48], [49]. In the absence of selection a population attains a stable age-distribution. Selection will alter *l_x_* and *m_x_* schedules and will rip a population out of a stable age-distribution. Demographic processes will tend to restore a stable age-distribution and with continued selection a tug of forces will ensue. Models of intense selection in age-structured populations [49] show that age-structure and age-dependency of selection can either increase or decrease the intensity of selection. In particular, if selective mortality hits reproductive ages (as is true here) age-structure may intensify selection. Therefore, there is hardly reason to think that age-structure will alleviate the threat of collapse. However, this remains to be investigated.

Fisheries-induced selection is most often considered size-selective mortality [12] directly targeting specific phenotypes. Here, however, selection is indirect. Fisheries are to a very large extent conducted in shallow rather than deep water [26] (Figure S5) and, therefore, fishing mortality will generate selection against genotypes adapted to shallow water although there is no direct targeting of specific phenotypes. There are general lessons in this for population and conservation genetics that changes in habitat can lead to intense selection even if the mortality is non-selective in the habitat in which it occurs. Sequence variation studies show that the *Pan* I *A* and *B* alleles are ancient predating the split of Atlantic cod from its sister species, the Pacific walleye pollock *Theragra chalcogramma* [15], [16]. Such studies provide a deep window into the species past evolutionary and selective history. However, here we observe a steep allele frequency gradient with depth and intense fisheries-induced selection. These factors clearly would influence measures of population differentiation such as *F*_ST_. We, therefore, question the use of *Pan* I and other loci under such intense selection as markers for analysis of population breeding structure due to reduced gene flow [18], [19], particularly if depth, the confounding of depth and geographic location, and habitat-specific fishing pressures are not controlled for. In particular, we question the practice of combining results from strongly selected loci such as *Pan* I with variation of supposedly neutral microsatellite loci to study breeding structure [18], [19] due to population isolation. The strongly selected loci will drive the overall measure of differentiation even in the face of considerable gene flow. Fisheries-induced selection can cause differentiation of *Pan* I among local groups and using that differentiation to argue for local population structure and special management of local populations may be circular.

There is a great need to understand selection and local molecular adaptation and how fisheries can indirectly and inadvertently generate intense selection as in this case. Conservation measures such as conserving large fish [13] would not be enough if they failed to protect genetic variants adapted to local niches.

### Fisheries-induced changes and evolution of life-history traits

Data on catch per unit effort are difficult to interpret as indicators of population abundance [28]. For the Icelandic cod fishery total catch is highly correlated with TAC and increases and decreases with it. Total catch always exceeds the TAC and thus the fishing mortality is greater than assumed with the recommended TAC. As expected fishing effort increases with catch and TAC and so does catch per unit effort. As catch diminishes for some gear, such as gill net, effort is also reduced and at a low total catch the catch per unit effort may increase again. Other gear behave differently. For long line in particular, which lately is taking a larger share of the total catch, both catch and effort have increased and stayed high. There are signs of changes in catchability [31], [50] and increased fishing pressure and effort as has been observed in the decline and collapse of other cod populations [11], [31].

Rapid changes in maturation preceded the collapse of the northern cod at Newfoundland [6]. Similarly we have indications of maturation changes occurring in Icelandic cod. The caveat is that we have not studied differential maturation of the sexes or potential effects of geographic location (e.g. north vs. south [19]) or environment and we are using averages as data. We interpret the trends in estimated probabilistic maturation reaction norms to mean that the sigmoid (logistic) maturation curves on length (and/or age) are changing. As their inflection points are pushed towards shorter lengths or lower ages they are also changing shape, becoming a stepped function. Probabilistic maturation reaction norms are useful for assessing genetic changes in the presence of environmental variation and phenotypic plasticity [32]. We, therefore, hypothesize that these are selective changes. Small and young fish may be evolving to delay reproduction while larger and older fish evolve to mature earlier and the fish become mature in a narrower window of length (or age). Overall, therefore, we have signatures for Icelandic cod of changes in effort and in life-history traits that are comparable to changes observed in the collapse of other cod stocks.

Olsen et al. [6] cautioned that “Although eroding maturation reaction norms can thus signal extreme exploitation pressures, they are not to be misinterpreted as signs of imminent stock collapse. But exploitation pressures so strong that they overturn a species' natural pattern of life-history adaptation certainly ought to be cause for concern.” Our study certainly appears to meet the criteria for concern. The strength of selection imposed by the fishery in Iceland is extremely high with selection coefficients of 92% and 73% against *AA* and *AB* genotypes respectively. This is in the high end in the distribution of known selection coefficients [51], [52]. The life-history changes coupled with the *Pan* I changes are perhaps even more dramatic than that documented for the northern cod [6].

### Future of population and fishery

We hypothesize that a collapse of the fishery is imminent if *Pan* I genotypic frequencies change as predicted. This hypothesis is supported also by changes in life-history. Considering fate of the fishery, deep-water fish are harder to catch and as they increase in frequency the fishery may become commercially in-viable. Fishing mortality in the preferred habitat would then cease before exterminating the *A* allele and fitness would revert back to natural values. Under that scenario selection pressures will diminish and the *A* allele may not go extinct completely which presumably would help subsequent recovery [6]. An alternative, and more likely, scenario is contraction of habitat use from tertiary and secondary to the most suitable primary habitat as a density-dependent response similar to the collapse of the northern cod stock in Newfoundland [53] and of the North Sea cod [54]. Fishers will go after smaller and smaller but equally dense clusters of fish in the primary habitat that still allow a profitable commercial fishery until the shallow water fish disappear from all habitats. Fisheries-induced selection at the *Pan* I locus may have contributed to the collapse of the northern cod and other threatened cod stocks. If fishing mortality causes large decreases in frequencies of the *A* allele it is unlikely to revert quickly back to previous values after fishing ceases. The fact that we see rapid changes in frequency means that back selection for *A* from natural causes clearly is much lower than the intense selection against *A* caused by the fishing mortality. Also, the lack of changes among fish greater than eight years of age (Figure 6) shows no evidence for back selection. Therefore, upon collapse the fishery would take a longer time to recover than it takes to collapse [11]. With *AA* and *AB* fish decimated by fishery we can inquire whether *BB* fish could invade the shallow-water niche and support a commercial fishery. This is unlikely as *BB* fish are deep-water adapted types [46]. We have found no evidence for historical separation or population structure by depth. However, to the extent that such a structure exists with limitation on interbreeding between shallow water and deep water fish a collapse would be more rapid and its effects would be harder to reverse.

Current management strategies of Icelandic cod are based on a catch limitation system where each vessel is allowed a certain share of the total allowable catch (TAC). Annual TACs are based on scientific assessment of state of fish stocks and ecosystem condition, but have been reduced lately. In addition, special measures for protecting small fish and the ecosystem are implemented. Thus relevant areas may be closed for short periods, if the percentage of small fish or by-catch exceed set limits. Additionally, cod spawning grounds are closed annually at the height of the spawning season in March and April to protect spawning fish. Apparently, however, these measures do not protect shallow-water fish as selection is similarly affecting all year classes that entered our samples (Figure 5). Furthermore, some current management measures may actually intensify selection. For example, during the March/April stop, deep-water *BB* fish move to shallow waters to spawn [46] (Table 1) whereupon they return to deep water and relative safety from fishing mortality. On re-opening, fishing mortality will hit the *AA* and *AB* fish that stay in shallow-water. Thus without knowledge of local adaptation good conservation intentions may exacerbate the problem.

### Averting collapse with no-take reserves

We consider that our study meets criteria for concern that the Icelandic cod stock is imperiled. Can anything be done to avert collapse? Upon collapse of the northern cod of Newfoundland the Canadian government imposed a moratorium on fishing [11]. Such a drastic measure if imposed in Iceland doubtless would avert collapse. Alternatively management measures that shifted fishing from shallow-water to deep-water or measures that distributed fishing effort evenly over all depth ranges by controlling fishing by different gear also could possibly help avert collapse. However, we consider that such strategies would be difficult to implement. Alternatively we speculate and suggest that it may be possible to avert collapse by adopting a different strategy of removing selection pressures against shallow-water adapted *AA* and *AB* fish. This highlights the use of evolutionary thinking for management and conservation issues. Given that current practices are ineffective in protecting shallow-water adapted fish, we suggest that immediate action is required. We suggest that establishment of large no-take marine reserves that range from the shoreline down to the very deep waters of at least 500 meters or more would protect all genotypes. In the case of Icelandic cod an obvious area is Selvogsbanki and Faxafloi, the main spawning grounds in the Southwest [26], 55. Additional areas would be the shallow-waters in the Northeast which were closed for some years with good results but subsequently re-opened [56]. The advantage of no-take reserves would be to relieve selection pressures against the shallow-water adapted *AA* and *AB* fish. Although there are gaps in our knowledge of no-take reserves [57], [58] we predict standard benefits of spillover of adults from prime into secondary and tertiary habitat and export of pelagic eggs and larvae that will ultimately benefit the fishery [59], [60].

## Materials and Methods

### Sampling and measurement

To assess temporal and spatial variation in *Pan* I frequencies we sampled cod measured and aged at all predetermined sampling stations during the Marine Research Institute spring spawning surveys in 2005, 2006, and 2007 in eight of nine divisions revised from definitions in the METACOD project [19], [61] (Figure S2). At each station a set with 12 gill nets of alternating six to nine inch mesh size made with mono-filament or multi-filament yarn was laid out. The sets stayed in place for at least 12 hours. Each net was 50 meters long and a set of 12 thus was 600 m long. The height of nets was 50–60 meshes or about 12–15 m. In the steep-slope deep waters off the South coast (Figure S2) a double set of 24 gill nets 1200 m long was laid out. A set of gill nets thus could cover a range of space and depths. We used mean location and mean depth in the analysis. We similarly took stratified random samples of stations taken during the MRI fall ground fish surveys in 2004, 2005, and 2006.

From each net up to 25 fish were taken for measurement of various individual traits. Otoliths were taken for age determination from a single fish from each net and a sample of gill tissue was taken for genetic analysis from these and preserved in 96% ethanol. The year class (cohort) of a fish was determined from the sample year and age read from otolith. Based on our sampling design most year classes entered our sampling for three consecutive ages except the very recent and old year classes which entered for one or two ages.

Commercial catches and effort for 1997–2007 by different gear were obtained from logbook data and from official statistics. Effort of bottom trawl is trawling time in minutes, effort of Danish seine is number of throws, effort of gill net is number of sets of nets, effort of hand line is number of hours at sea, and effort of long line is number of lines times number of hooks per line. Log book results on effort and catch per unit effort for most of the gear have also been presented in figures 9.3.1 and 9.3.2 in [62].

### Molecular analysis

The *Pan* I locus of Atlantic cod has two alleles, *A* and *B*, defined by the presence or absence of a *Dra*I restriction site [63]. We used a proteinase K digestion/chelex 100 method [64] for DNA isolation from tissue. We used primers 3 and 20 [15] to amplify a 489 base pair fragment of the *Pan* I gene and digested that with *Dra*I to reveal diagnostic bands of the three genotypes on an agarose gel [63]. Altogether we genotyped over 8100 individuals.

### Estimating probabilistic maturation reaction norms

To estimate probabilistic maturation reaction norms [32] we used methods for estimating probabilities of maturing from maturity ogives [33], [34]. We used data on sexual maturity at age in Marine Research Institute spring surveys (Table 3.1.4 in [35]) and mean length and age. We estimated maturity ogives *o*, probability of being mature at mean length and age, with a generalized linear model (glm) logistic regression: 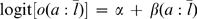 with sample sizes as weights. The data are mean maturity and mean length *l̅* at age *a* and thus fitting a full model [34] is not possible. Instead the interaction term *a*:*l̅* takes into account potential non-linearity of age and length. Following Barot et al. 33, 34 probability of becoming mature *m* was estimated as 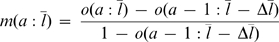
To parameterize the reaction norms we fitted a logistic model of logit(*m*) on mean length or age and calculated the quantiles of length and age at 5%, 50% and 95% *m* using the dose.p function of the MASS library under R [65].

### Statistics and fitness estimation

We mostly used R [66] and various in house functions and packages under R for statistical and genetic analysis. In particular we used the LATTICE package [67], [68] that implements Trellis graphics in R, the MGCV package [69], [70] for fitting generalized additive models (gam) and the HIERFSTAT package [71] for R that implements an algorithm [72] for estimating *F*-statistics at any level of a nested or hierarchical structure.

Fitness is considered a weight, *U*, that transforms a genotypic frequency at one age into a frequency at another and higher age. To estimate the weight we took ratios of genotypes frequencies at two ages. We used the generalized additive model (gam) fit (Figure 6), with a quasibinomial link function to model overdispersion, to predict genotypic frequencies and approximate 95% confidence intervals at 4 and 8 years of age (Table 6). We took the ratio of these predictions to estimate the weights. Furthermore, we took the ratios of upper to lower and lower to upper confidence intervals for predictions of best-case and worst-case scenarios. Relative fitnesses, *W_i_*, are the weights scaled to the most fit *Pan* I *BB* genotype.

In supplementary material (Table S2) we also took the pooled observed frequencies among 3 and 4 year old (entering the fishery; “pre-selection”) and among 8–13 year old (ages at which frequencies do not change much and the brunt of the fishery is over; “post-selection”) to estimate the weights and standard errors based on variance of ratios. In Table S3 we used median genotypic frequencies at age within the various year classes to estimate weights as yearly transitions. Assuming independent action of weights in time we multiplied the yearly weights to get an overall weight.

We used our estimated fitness values to plug into a constant-fitness viability model [73] and assuming non-overlapping generations [49]

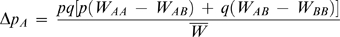
for predicting allele and genotypic frequency changes at *Pan* I. The effect of selection and age-structure [48], [49] was not considered here.

## Supporting Information

Figure S1 **Generalized additive model smooth fit of frequency of *A* allele on depth of sampling.** Shaded region represents two standard errors above and below fit; points are partial residuals; the gam smooth was fitted using a quasibinomial link function to model overdispersion; estimated degrees of freedom edf = 3.2; approximate significance of smooth term *F = 47.95*, *P≪0.001* with 51.4% of deviance explained.
(1.17 MB TIF)

Figure S2 **Sampling stations for Atlantic cod on a map of Iceland.** Icelandic Marine Research Institute spring spawning surveys conducted in 2005, 2006, and 2007. Color coded divisions are based on revised METACOD definitions [19], [61]. No samples were taken from division 6 (not shown on map).
(2.50 MB TIF)

Figure S3 **Sampling stations for Atlantic cod conditioned on 25 m depth classes on a map of Iceland.** Icelandic Marine Research Institute spring spawning surveys in 2005, 2006, and 2007. Color coded divisions as in Supporting Figure S2.
(2.88 MB TIF)

Figure S4 **Conditional densityplot of catch of Atlantic cod at Iceland on age by year class.** Landings in numbers (millions). Age based on length at age relationships. Based on data from Table 3.1.6 in [27].
(2.59 MB TIF)

Figure S5 **Density plot of catch on depth (m) by year for different gear.** Data are from log book records.
(0.72 MB TIF)

Figure S6 **Total and proportional catch by gear in different years.** Total catch in tons and proportion of total by gear. Catch data for 1992–2007 are official statistics from Statistics Iceland http://www.statice.is. Data for 2008 are provisional data from Directorate of Fisheries http://www.fiskistofa.is/en.
(0.89 MB TIF)

Figure S7 **Total catch, issued total allowable catch, TAC, and recommended TAC.** Total catch (black solid dots, solid lines) of Atlantic cod at Iceland, government issued total allowable catch, TAC (red open dots, dashed lines), an Marine Research Institute recommended total allowable catch, recommended TAC (blue pluses and dotted lines) on year. Based on data from Table 2.1.1 in [27].
(0.65 MB TIF)

Figure S8 **Allelic and genotypic frequencies on year class conditioned on age among spring spawning Atlantic cod.** Frequency of *A* allele, *pA* (top panel row), and frequencies of *AA*, *AB*, and *BB* genotypes, *fAA*, *fAB*, and *fBB* (panel rows 2–4 respectively). Panels represent ages arranged from young to old from left to right in each row. Points represent observed frequencies; lines represent linear regression of frequency on yearclass.
(2.79 MB TIF)

Table S1 **A table summarizing the key points of the supporting information data.**
(0.05 MB ZIP)

Table S2 **Genotypic frequencies pooled pre and post selection, weigths and fitnesses.**
(0.03 MB ZIP)

Table S3 **Fitness based on weights as ratios of median genotypic frequency changes between years within year class.**
(0.03 MB ZIP)

## References

[1] Kurlansky M (1997). Cod: A Biography of the Fish That Changed the World.

[2] Rijnsdorp AD (1993). Fisheries as a large-scale experiment on life-history evolution—disentangling phenotypic and genetic effects in changes in maturation and reproduction of North-Sea plaice, *Pleuronectes platessa* L.. Oecologia.

[3] Hutchings JA (2004). The cod that got away.. Nature.

[4] Law R (2000). Fishing, selection, and phenotypic evolution.. ICES J Mar Sci.

[5] Conover DO (2000). Darwinian fishery science.. Mar Ecol Prog Ser.

[6] Olsen EM, Heino M, Lilly GR, Morgan MJ, Brattey J (2004). Maturation trends indicative of rapid evolution preceded the collapse of northern cod.. Nature.

[7] Olsen EM, Lilly GR, Heino M, Morgan MJ, Brattey J (2005). Assessing changes in age and size at maturation in collapsing populations of Atlantic cod (*Gadus morhua*).. Can J Fish Aquat Sci.

[8] Kuparinen A, Merilä J (2007). Detecting and managing fisheries-induced evolution.. Trends Ecol Evol.

[9] Jørgensen C, Enberg K, Dunlop ES, Arlinghaus R, Bouka DS (2007). Managing evolving fish stocks.. Science.

[10] Conover DO (2007). Nets versus nature.. Nature.

[11] Hutchings JA (2000). Collapse and recovery of marine fishes.. Nature.

[12] Swain DP, Sinclair AF, Hanson JM (2007). Evolutionary response to size-selective mortality in an exploited fish population.. Proc R Soc Ser B.

[13] Law R (2007). Fisheries-induced evolution: present status and future directions.. Mar Ecol Prog Ser.

[14] Hutchings JA, Fraser DJ (2008). The nature of fisheries- and farming-induced evolution.. Mol Ecol.

[15] Pogson GH, Mesa K (2004). Positive Darwinian selection at the Pantophysin (*Pan* I) locus in marine Gadid fishes.. Mol Biol Evol.

[16] Pogson GH (2001). Nucleotide polymorphism and natural selection at the Pantophysin (*Pan* I) locus in the Atlantic cod, *Gadus morhua* (L.).. Genetics.

[17] Karlsson S, Mork J (2003). Selection-induced variation at the pantophysin locus (*Pan* I) in a Norwegian fjord population of cod (*Gadus morhua* L.).. Mol Ecol.

[18] Sarvas TH, Fevolden SE (2005). Pantophysin (*Pan* I) locus divergence between inshore v. offshore and northern v. southern populations of Atlantic cod in the north-east Atlantic.. J Fish Biol.

[19] Pampoulie C, Ruzzante DE, Chosson V, Jörundsdóttir TD, Taylor L (2006). The genetic structure of Atlantic cod *Gadus morhua* around Iceland: Insights from microsatellites, the *Pan* I locus, and tagging experiments.. Can J Fish Aquat Sci.

[20] Stockwell CA, Hendry AP, Kinnison MT (2003). Contemporary evolution meets conservation biology.. Trends Ecol Evol.

[21] Wahlund S (1928). Zuzammensetzung von populationen und korrelationserscheinungen vom standpunkt der vererbungslehre aus betrachtet.. Hereditas.

[22] Johannesson K, Tatarenkov A (1997). Allozyme variation in a snail (*Littorina saxatilis*)–-deconfounding the effects of microhabitats and gene flow.. Evolution.

[23] de Meeûs T, Goudet J (2007). A step-by-step tutorial to use HierFstat to analyse populations hierarchically structured at multiple levels.. Infect, Genet Evol.

[24] Fisher R (1970). Statistical Methods for Research Workers.

[25] Case RAJ, Hutchinson WF, Hauser L, Oosterhout CV, Carvalho GR (2005). Macro- and micro-geographic variation in pantophysin (*Pan* I) allele frequencies in NE Atlantic cod *Gadus morhua*.. Mar Ecol Prog Ser.

[26] Begg GA, Marteinsdottir G (2003). Spatial partitioning of relative fishing mortality and spawning stock biomass of Icelandic cod.. Fish Res.

[27] Anonymous (2007). State of marine stocks in Icelandic waters 2007/2008. Prospects for the quota year 2008/2009. Technical Report nr. 138, Marine Research Institute, Reykjavík, Iceland..

[28] Maunder MN, Sibert JR, Fonteneau A, Hampton J, Kleiber P (2006). Interpreting catch per unit effort data to assess the status of individual stocks and communities.. ICES J Mar Sci.

[29] Anonymous (2000). State of marine stocks in Icelandic waters 1999/2000. Prospects for the quota year 2000/2001. Technical Report nr. 75, Marine Research Institute, Reykjavík, Iceland..

[30] Anonymous (2001). State of marine stocks in Icelandic waters 2000/2001. Prospects for the quota year 2001/2002. Technical Report nr. 80, Marine Research Institute, Reykjavík, Iceland..

[31] Myers RA, Hutchings JA, Barrowman NJ (1996). Hypothesis for the decline of cod in the North Atlantic.. Mar Ecol Prog Ser.

[32] Heino M, Dieckmann U, Godø OR (2002). Measuring probabilistic reaction norms for age and size at maturation.. Evolution.

[33] Barot S, Heino M, O'Brien L, Dieckmann U (2004). Estimating reaction norms for age and size at maturation when age at first reproduction is unknown.. Evol Ecol Res.

[34] Barot S, Heino M, OBrien L, Dieckmann U (2004). Long-term trend in the maturation reaction norm of two cod stocks.. Ecol Appl.

[35] Anonymous (2007). State of marine stocks in Icelandic waters 2006/2007. Prospects for the quota year 2007/2008. Technical Report nr. 126, Marine Research Institute, Reykjavík, Iceland..

[36] Árnason E (2004). Mitochondrial cytochrome *b* DNA variation in the high fecundity Atlantic cod: Trans-Atlantic clines and shallow gene-genealogy.. Genetics.

[37] Waples RS (1998). Separating the wheat from the chaff: Patterns of genetic differentiation in high gene flow species.. Heredity.

[38] Nielsen EE, Hansen MM, Meldrup D (2006). Evidence of microsatellite hitch-hiking selection in Atlantic cod (*Gadus morhua* L.): Implications for inferring population structure in nonmodel organisms.. Mol Ecol.

[39] Westgaard J, Fevolden S (2007). Atlantic cod (*Gadus morhua* L.) in inner and outer coastal zones of northern Norway display divergent genetic signature at non-neutral loci.. Fish Res.

[40] Pogson GH, Fevolden S (2003). Natural selection and the genetic differentiation of coastal and Arctic populations of the Atlantic cod in northern Norway: a test involving nucleotide sequence variation at the Pantophysin (*Pan* I) locus.. Mol Ecol.

[41] Sarvas TH, Fevolden S (2005). The scnDNA locus *Pan* I reveals concurrent presence of different populations of Atlantic cod (*Gadus morhua* L.) within a single fjord.. Fish Res.

[42] Lewontin RC, Cockerham CC (1959). The goodness-of-fit test for detecting natural selection in random mating populations.. Evolution.

[43] Wallace B (1958). The comparison of observed and calculated zygotic distributions.. Evolution.

[44] Prout T (1965). The estimation of fitness from genotypic frequencies.. Evolution.

[45] Robertson A (1965). The interpretation of genotypic ratios in domestic animal populations.. Anim Prod.

[46] Pampoulie C, Jakobsdóttir KB, Marteinsdóttir G, Thorsteinsson V (2007). Are vertical behaviour patterns related to the Pantophysin locus in the Atlantic cod (*Gadus morhua* L.)?. Behav Genet.

[47] Pálsson ÓK, Thorsteinsson V (2003). Migration patterns, ambient temperature, and growth of Icelandic cod (*Gadus morhua*): evidence from storage tag data.. Can J Fish Aquat Sci.

[48] Charlesworth B (1994). Evolution in Age-Structured Populations.

[49] Galvani AP, Slatkin M (2004). Intense selection in an age-structured population.. Proc R Soc Lond Ser B.

[50] Swain DP, Nielsen GA, Sinclair AF, Chouinard GA (1994). Changes in catchability of Atlantic cod (*Gadus morhua*) to an otter-trawl fishery and research survey in the southern Gulf of St Lawrence.. ICES J Mar Sci.

[51] Endler J (1986). Natural Selection in the Wild.

[52] Kingsolver JG, Hoekstra HE, Hoekstra JM, Berrigan D, Vignieri SN (2001). The strength of phenotypic selection in natural populations.. Am Nat.

[53] Hutchings JA (1996). Spatial and temporal variation in the density of northern cod and a review of hypotheses for the stock's collapse.. Can J Fish Aquat Sci.

[54] Blanchard JL, Mills C, Jennings S, Fox CJ, Rackham BD (2001). Distribution-abundance relationships for North Sea Atlantic cod (*Gadus morhua*): observation versus theory.. Can J Fish Aquat Sci.

[55] Marteinsdottir G, Gunnarsson B, Suthers IM (2000). Spatial variation in hatch date distributions and origin of pelagic juvenile cod in Icelandic waters.. ICES J Mar Sci.

[56] Schopka SA (2007). Area closures in Icelandic waters and the real-time closure system. A historical review. Technical Report Technical Report nr. 133, Marine Research Institute, Reykjavík, Iceland..

[57] Sale PF, Cowen RK, Danilowicz BS, Jones GP, Kritzer JP (2005). Critical science gaps impede use of no-take fishery reserves.. Trends Ecol Evol.

[58] Ewers RM, Rodrigues AS (2008). Estimates of reserve effectiveness are confounded by leakage.. Trends Ecol Evol.

[59] Roberts CM, Bohnsack JA, Gell F, P HJ, Goodridge R (2001). Effects of marine reserves on adjacent fisheries.. Science.

[60] Gell FR, Roberts CM (2003). Benefits beyond boundaries: the fishery effects of marine reserves.. Trends Ecol Evol.

[61] Jónsdóttir IG, Campana SE, Marteinsdottir G (2006). Otolith shape and temporal stability of spawning groups of Icelandic cod (*Gadus morhua* L.).. ICES J Mar Sci.

[62] ICES (2008).

[63] Fevolden SE, Pogson GH (1997). Genetic divergence at the Synaptophysin (*Syp* I) locus among Norwegian coastal and north-east Arctic populations of Atlantic cod.. J Fish Biol.

[64] Walsh PS, Metzger DA, Higuchi R (1991). Chelex 100 as a medium for simple extraction of DNA for PCR-based typing from forensic material.. BioTechniques.

[65] Venables WN, Ripley BD (2002). Modern Applied Statistics with S.

[66] R Development Core Team (2007).

[67] Sarkar D (2007). lattice: Lattice Graphics..

[68] Sarkar D (2008). Lattice: Multivariate Data Visualization with R.

[69] Wood S (2006). Generalized Additive Models: An Introduction with R.

[70] Wood S (2008). Fast stable direct fitting and smoothness selection for generalized additive models.. J R Stat Soc Ser B.

[71] Goudet J (2005). HIERFSTAT, a package for R to compute and test hierarchical *F*-statistics.. Mol Ecol Notes.

[72] Yang R (1998). Estimating hierarchical *F*-statistics.. Evolution.

[73] Hedrick PW (2005). Genetics of Populations.

[74] Björnsson H, Sólmundsson J, Kristinsson K, Steinarsson BÆ, Hjörleifsson E (2007). Stofnmæling botnfiska á Íslandsmidum (SMB) 1985–2006 og stofnmæling botnfiska ad haustlagi (SMH) 1996–2006. undirbúningur, framkvæmd og helstu nidurstödur. Technical Report nr. 131, Hafrannsóknastofnunin. Marine Research Institute, Reykjavík, Iceland..

