# Supplementary material for: Intense Habitat-Specific Fisheries-Induced Selection at the Molecular Pan I Locus Predicts Imminent Collapse of a Major Cod Fishery
Source: PLoS One. 2009 May 27;4(5):e5529. doi: 10.1371/journal.pone.0005529 (PMC2682699; doi:10.1371/journal.pone.0005529)
Supplement: Table S1 — A table summarizing the key points of the supporting information data. (0.05 MB ZIP) [file pone.0005529.s009.zip › TableS1/TableS1.pdf]

Table S1. A table summarizing the key points of the supporting information data.

Supporting	Key points
**Figure S1**	The figure shows the gam regression of the logit of frequency ($\log p/q$) on depth. It shows the statistical support for the regression with standard errors and residuals. It supplements Figure 1 of the paper which, on a more familiar scale, shows the allele frequency p on depth.
**Figure S2**	The figure shows the locations of sampling stations on a map of Iceland. Sampling stations are color coded based on revised METACOD definitions [19,61]. The METACOD divisions are based on oceanographic features, currents and such. These divisions are used in testing for population differentiation in Tables 2, 3, and 4 of the paper.
**Figure S3**	The figure presents the evidence for confounding of depth and geographic location (division). In particular, shallow water stations are found in the north and north-east and deep-water stations in the south. The effects of confounding are studied in Tables 3 and 4 in the paper.
**Figure S4**	The figure shows that the brunt of the fishing is carried by 5–7 year old fish. Eight year old fish is severly reduced in numbers and nine year olds are all but fished out. Among the very old year classes old fish are only a tiny proportion of the catch. Among the recent year classes young fish are entering the fishery, become an increasing proportion and, with age, quickly become a decreasing proportion.
**Figure S5**	The figure shows that fishing is concentrated in shallow water. The density of fishing by long line has shifted to shallower water in more recent years. At the same time long line is taking an increasing proportion of total catch as Figure S6 and Figure 3 in paper show. Thus recent changes in fishing have also been directed at shallow water.
**Figure S6**	The figure shows total catch by gear and proportional catch. There is trend towards reduced catch during the past ten years. The reduction in catch in the population crash of 1995 is evident.. The proportion of the catch taken by different gear also has changed. Proportion of catch taken by bottom trawl, which is distributed over the greatest depth range, has decreased and proportion of catch taken by gear mainly targeting shallow water, long line in particular, has increased.

Continued on next page

Table S1. Continuation.

Supporting	Key points
**Figure S7**	Catch is regulated by government issued quotas. The figure shows the complex relationships of catch, issued TAC's and recommended TAC's based on scientific assessment of the stock. Although there are large fluctuations there is a downward trend in all numbers. This figure supplements Figure 3 in the paper which presents data on catch, effort and catch per unit effort
**Figure S8**	The figure shows allelic and genotypic frequencies on year class conditioned on age. It complements Figure 5 in the paper which shows allelic and genotypic frequencies on age conditioned on year class. The intense fisheries-induced selection is expected to reduce the frequency of the A allele. There is a trend for more recent year classes to have lower frequencies of the A allele as expected from the intense selection.
**Table S2**	The table presents fitness estimates by calculating weights U_i as ratios of frequencies of young (3–4 year olds entering the fishery as "preselection") and old (8–13 year olds, "post-selection"). The estimates are based on observed numbers and standard errors are based on the variance of ratios. Based on a slightly different method from that in Table 6 in the paper the results are nevertheless similar. The fitness estimates from this table are used for making predictions of changes shown in top right panel of Figure 7 in the paper.
**Table S3**	The table presents weights U_{ij} as ratios of frequencies at one year age intervals (j). Assuming independent action of the selective forces we multiply weights for ages 5–11 to calculate overall weights. Fitness estimated by this method are the most extreme. Predicted changes using these fitnesses (not shown) are even more drastic than the worst-case scenario presented in Table 6 and Figure 7 of the paper.
