# Supplementary material for: Intense Habitat-Specific Fisheries-Induced Selection at the Molecular Pan I Locus Predicts Imminent Collapse of a Major Cod Fishery
Source: PLoS One. 2009 May 27;4(5):e5529. doi: 10.1371/journal.pone.0005529 (PMC2682699; doi:10.1371/journal.pone.0005529)
Supplement: Table S2 — Genotypic frequencies pooled pre and post selection, weigths and fitnesses. (0.03 MB ZIP) [file pone.0005529.s010.zip › TableS2/TableS2.pdf]

Table S2. Genotypic frequencies pooled pre and post selection, weigths and fitnesses.

	AA			*AB*			*BB*			
Age	obs	rel	SE	obs	rel	SE	obs	rel	SE	Sum
3–4	81	0.57	0.04	46	0.33	0.04	14	0.10	0.00	141
8–13	123	0.24	0.02	197	0.39	0.02	191	0.37	0.02	511
U		0.42	0.05		1.18	0.16		3.76	0.98	
W		0.11	0.28		0.31	0.29		1.00	0.37	

Genotypic frequencies pooled among 3–4 ("pre-selection") and 8–13 years old ("post-selection"), weights, U, and fitness, W, relative to the most fit BB genotype. Respective standard errors are based on variance of ratios.
