# Supplementary material for: Intense Habitat-Specific Fisheries-Induced Selection at the Molecular Pan I Locus Predicts Imminent Collapse of a Major Cod Fishery
Source: PLoS One. 2009 May 27;4(5):e5529. doi: 10.1371/journal.pone.0005529 (PMC2682699; doi:10.1371/journal.pone.0005529)
Supplement: Table S3 — Fitness based on weights as ratios of median genotypic frequency changes between years within year class. (0.03 MB ZIP) [file pone.0005529.s011.zip › TableS3/TableS3.pdf]

Table S3. Fitness based on weights as ratios of median genotypic frequency changes between years within year class.

	Weights, U		
Age	U_{AA}	U_{AB}	U_{BB}
4	1.164	0.752	1.214
5	0.829	1.004	2.061
6	0.714	0.946	2.920
7	0.731	1.014	1.288
8	0.691	0.853	1.425
9	0.932	0.995	1.055
10	0.750	0.986	1.164
11	0.864	0.901	0.875
12	0.505	0.812	3.940
13	1.167	1.061	0.875
$\Pi_{j=5}^{11} U_{ij}$	0.181	0.726	11.873
W_i	0.015	0.061	1.0

Products of weights, $\Pi_{j=5}^{11} U_{ij}$, for ages 5–11. Fitness of genotypes, W_i, relative to the most fit BB genotype.
